# Immunogenicity of monovalent and multivalent subunit vaccines against SARS-CoV-2 variants in mice with divergent vaccination history

**DOI:** 10.1128/spectrum.02907-24

**Published:** 2025-07-17

**Authors:** Rui Wang, Yuan Lyu, Meng Chen, Lili Sun, Shaozheng Zhou, Yudi Cui, Juan Ma, Desheng Kong, Jianbo Lu, Xuefeng Li, Liangzhi Xie

**Affiliations:** 1 Beijing Engineering Research Center of Protein and Antibody, Sinocelltech Ltd. 623683 , Beijing, China; 2 Beijing Key Laboratory of Monoclonal Antibody Research and Development, Sino Biological Inc. 479665 , Beijing, China; 3 Cell Culture Engineering Center, Chinese Academy of Medical Sciences & Peking Union Medical College, Beijing, China; Emory University School of Medicine, Atlanta, Georgia, USA

**Keywords:** SARS-CoV-2, monovalent vaccines, multivalent vaccines, vaccine-induced immunity

## Abstract

There are challenges in selecting SARS-CoV-2 vaccine compositions, primarily due to divergent infection or vaccination history and immunological biases toward previous strains. In this study, we evaluated humoral immune responses induced by variant-based monovalent vaccines as booster shots in mice previously vaccinated with an ancestral strain-based vaccine with or without Omicron BA.5 exposure. Our data suggest that immunological biases toward earlier variants attenuated the potency of variant-based vaccines as a booster dose against subsequent variants, whereas a second booster dose mitigated this effect. Furthermore, in the context of vaccine-induced immunity, prior exposure to Omicron sublineages (e.g., BA.5) attenuated the effect of immunological biases toward earlier variants on the neutralizing potency against Omicron subvariants. In addition, the interval between vaccine doses should also be considered, as an immunologic plateau might occur after repeated vaccination. Furthermore, the XBB.1 monovalent vaccine and a tetravalent vaccine (SCTV01E-2) composed of pre-Omicron variant (Beta) and Omicron subvariants (BA.1, BQ.1.1, and XBB.1) showed comparable neutralizing potency against several Omicron sublineages (BA.1, BA.5, BQ.1.1, XBB.1, XBB.1.5, XBB.1.16, and EG.5) under divergent vaccination history, implicating that multivalent platforms could be explored as a flexible strategy if future strains diverge significantly from current variants.

**IMPORTANCE:**

Continuous evolution of SARS-CoV-2 variants has raised the need to optimize immunization regimens and update vaccine compositions to protect against the newly emerging variants in the context of repeated vaccination. The significance of this research is briefly summarized as follows:

1) Immunological biases toward earlier variants attenuated the potency of variant-based vaccines as a booster dose against subsequent variants, which can be mitigated by a second booster dose.

2) In the context of vaccine-induced immunity, a previous exposure to Omicron sublineages, such as BA.5, attenuated the influence of immunological biases toward earlier variants on the neutralizing potency against Omicron subvariants.

3) The interval between vaccine doses should be taken into account since an immunologic plateau might occur after repeated vaccination.

4) Multivalent vaccines, with epitope diversity, may theoretically enhance the magnitude and breadth of cross-neutralization responses, thereby providing a buffer for unpredictable future variants.

## INTRODUCTION

Severe acute respiratory syndrome coronavirus 2 (SARS-CoV-2), the causative agent of coronavirus disease 2019 (COVID-19), has caused more than 7 million deaths worldwide since the beginning of the pandemic (1, 2). In response, in the context of COVID-19 vaccination campaigns, over 13 billion vaccine doses have been administered worldwide as of March 2023 (3). Mass vaccination is an effective public health intervention for curbing the ongoing pandemic. Nevertheless, the continuous evolution of SARS-CoV-2 has led to breakthrough infections, primarily due to the emergence of the Omicron BA.1 and subsequent sublineages (4–6). Worryingly, the newly emerged Omicron sublineages have severely compromised the protective efficacy of COVID-19 vaccines, leading to breakthrough infections, re-infections, and sustained transmission (6). Therefore, it was an urgent need to update vaccine components against newly circulating viruses and generate cross-protective immunity against future variants (7).

Based on antigenic divergence and the potential for immune bias induced by the SARS-CoV-2 ancestral strain, WHO has recommended excluding the original variant antigen from future vaccine updates (2). Instead, XBB variants should be included to improve immunity against newly emerging variants (8). EMA and FDA have approved XBB-based monovalent vaccines for populations aged 6 months and older (2, 7, 9).

The selection of vaccine components presents challenges primarily due to various immunity histories and immune biases, as well as uncertainty about future circulating viruses (10). First, a substantial portion of the population has undergone divergent COVID-19 vaccinations (11). The efficacy of booster doses may vary depending on prior infection and vaccination history (12). Second, in the context of immune bias toward ancestral strain, the breadth and capacity of immune responses against drifted variants might be diminished (13, 14). In addition, as SARS-CoV-2 continues to evolve, there is a widespread awareness of uncertainty about dominant strains in the future (15, 16). Therefore, exploring the impact of vaccination in populations with different exposure histories might provide valuable insights into the dynamic relationship between newly emerging strains, vaccination strategies, and the sub-population immune characteristics (17).

This study elucidates the breadth and durability of humoral immune responses elicited by monovalent vaccines based on either pre-Omicron variant (Beta) or the Omicron sublineages (BA.5, BQ.1.1, or XBB.1), when administered as booster doses in mice previously vaccinated with ancestral strain-based vaccine, either with or without Omicron BA.5 exposure. Meanwhile, a comparative study between an XBB.1 monovalent vaccine and a tetravalent vaccine containing Beta, BA.1, BQ.1.1, and XBB.1 (designated SCTV01E-2) was carried out under vaccine-induced immunity.

## MATERIALS AND METHODS

### Vaccine constructs and design

The monovalent vaccines of various strains (Beta, BA.5, BQ.1.1, and XBB.1) and SCTV01E-2 (tetravalent vaccine, Beta: BA.1: BQ.1.1: XBB.1 = 1: 1: 2: 2) were recombinant protein vaccines. SARS-CoV-2 spike protein extracellular domain (S-ECD) was fused to a T4 bacteriophage fibritin motif (i.e., T4 Foldon) to stabilize the conformation of the trimeric protein (18). Antigens were developed and produced by CHO cells based on mature technology and purification platforms. The detailed production and purification protocols were as previously described (19). The purified antigens were dissolved in the oil-in-water adjuvant SCT-VA02B and formulated into the vaccine product for subsequent research (20).

### Animal studies

Specific pathogen-free (SPF) female BALB/c or C57BL/6J mice (6–8 weeks old) were purchased from Beijing Vital River Laboratory Animal Technology Co., Ltd. The animals were fed a standard diet and maintained under a 12 h light/light cycle and a 12 h dark/light cycle. The temperature in the animal room was 20°C–25°C, and the humidity was 40%–70%. All animal studies were performed in accordance with protocols approved by the Institutional Animal Care and Use Committee (IACUC). All animal experiments were approved by the Laboratory Animal Welfare and Ethics Committee of the State Food and Drug Control (No. 2021 [B] 045 & 2022 [B] 039).

Mice were immunized with two doses of D614G monovalent vaccine 2 weeks apart, followed by the administration of BA.5-based vaccine (mimicking infection or vaccination with the BA.5 variant), and one or two doses of monovalent vaccines against Beta, BA.5, BQ.1.1, and XBB.1, and SCTV01E-2 booster at each time point (as shown in the immunization scheme in Figures), respectively. The doses of primary immunization, BA.5 exposure, SCTV01E-2, and monovalent vaccine booster were all 1 µg/dose. The serum was collected 1 or 2 weeks after the last immunization to detect pseudoviruses neutralization activities against multiple SARS-CoV-2 variants (including D614G, Beta, BA.1, BA.5, BQ.1.1, XBB.1, XBB.1.5, XBB.1.16, EG.5, BA.2.86, JN.1, KP.2, KP.1.1, and JN.1.11.1).

### Pseudovirus neutralization assay

SARS-CoV-2 pseudoviruses encoding firefly luciferase gene were produced by Sinocelltech Ltd (Beijing, China), and the detailed construction method has been described previously (21, 22). In summary, mouse serum samples were heat-inactivated at 56°C for 30 min and then serially diluted. The diluted serum was then incubated with 200 TCID50 of pseudovirus per well at 37°C for 1 h and then co-cultured with Huh7 cells (20,000 cells per well) for approximately 20 h. Luciferase activity was assessed by measuring relative light units (RLU) using a CentroXS3 LB 960 Microplate Luminometer. Neutralization titers were defined as the 50% inhibitory concentration (NAT50_50_) and calculated as described above (19).

### Live virus microneutralization assay

Live virus microneutralization was conducted by the National Institute for Viral Disease Control and Prevention, Chinese Center for Disease Control and Prevention (Beijing, China). The detailed procedures were described previously (23, 24). In brief, serum samples collected from immunized mice were heat-inactivated at 56°C for 30 min and then serially diluted. The virus was diluted to a concentration of 100 CCID50/0.05 mL and mixed with the serum dilutions in a 96-well plate (CCID50: cell culture infectious dose 50%). The plate was incubated at 37°C for 1–2 h to allow the antibodies in the serum samples to neutralize the virus. Vero cells (10,000–15,000 cells per well) were subsequently added to the serum-virus mixture, and the plate was incubated at 37°C for 3–5 days. Virus titers were assessed using the Karber formula, and the neutralizing titers were determined by the dilution number of 50% protective condition (MNT_50_).

### Statistical analysis

Statistical analysis was performed using GraphPad Prism 8.0. An unpaired two-tailed t-test was used to compare two independent experimental groups. Data were presented as mean ± SD. *P* < 0.05 was considered statistically significant. No animals or data points were excluded from the analysis.

## RESULTS

### Neutralizing capacity of monovalent vaccines based on pre-Omicron variant (Beta) or Omicron sublineages (BA.5, BQ.1.1, or XBB.1) as booster doses following a primary series based on ancestral strain

**Fig 1:**
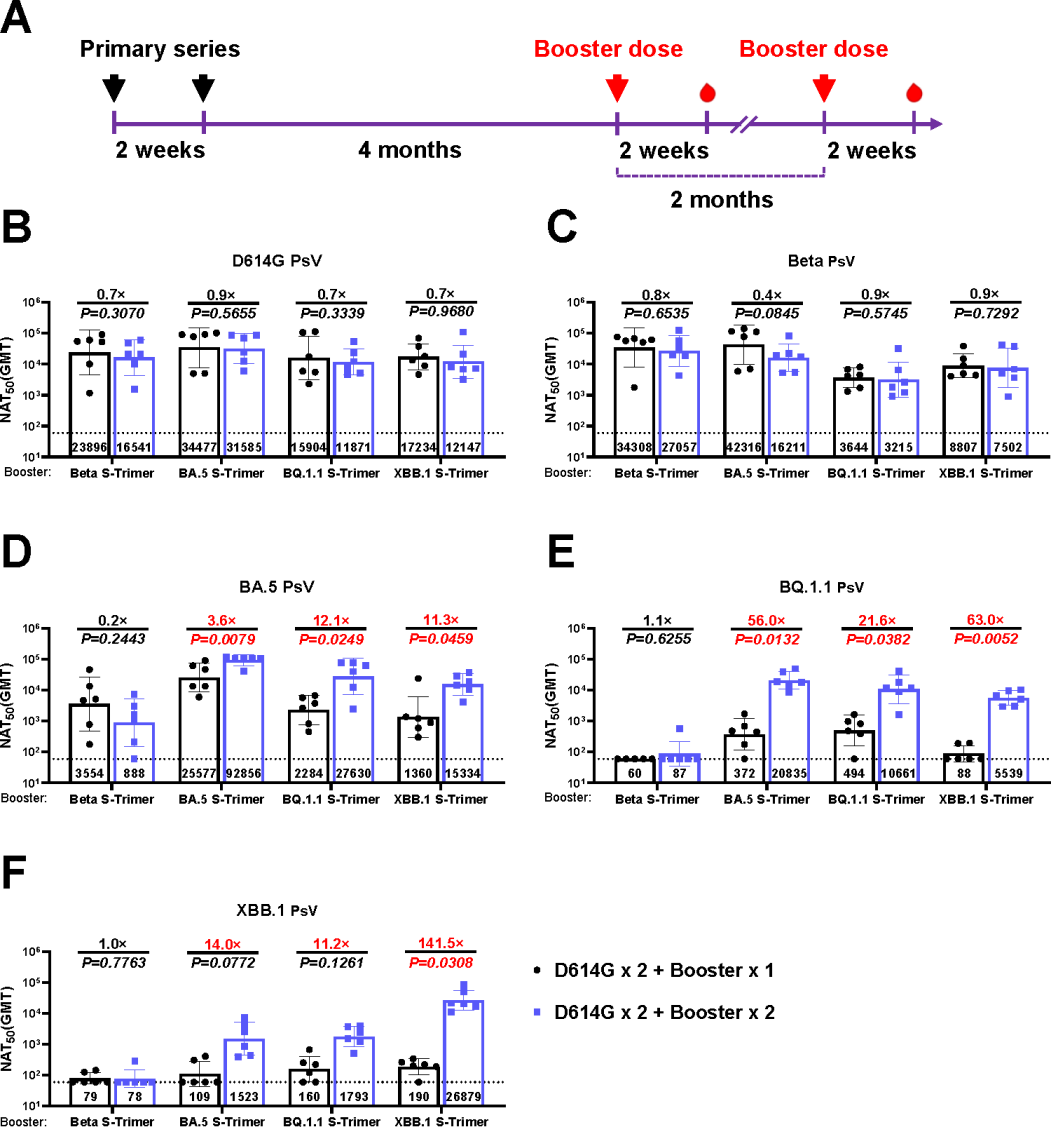
Neutralizing antibody responses of monovalent vaccines as one- or two-dose booster shots following priming series against the ancestral strain and Omicron subvariants in mice. (**A**) Schematic of the immunization design. Mice were intramuscularly administered two doses of D614G monovalent vaccine on Day 0 and Day 14. After 4 and 6 months, the monovalent vaccines (Beta, BA.5, BQ.1.1, or XBB.1) were injected into mice as one or two doses of booster shots. Serum neutralizing titers against PsV displaying D614G (**B**), Beta (**C**), BA.5 (**D**), BQ.1.1 (**E**), and XBB.1 (**F**) were measured. *P* values and fold change of neutralization were annotated above each bar, and geometric mean titers (GMTs) were labeled in the corresponding columns. Data were represented as mean ± SD, *n* = 5-6/group. Due to limited serum availability, 5 mice were included for the assessment of BQ.1.1 neutralization. Neutralizing capacity of monovalent vaccines based on pre-Omicron variant (Beta) or Omicron sublineages (BA.5, BQ.1.1, or XBB.1) as booster doses following ancestral strain-based primary series with or without subsequent BA.5 exposure.

To assess the immunogenicity of monovalent vaccines (based on trimeric spike protein of SARS-CoV-2 variants Beta, BA.5, BQ.1.1, or XBB.1) as booster shots, Balb/c mice were immunized with a two-dose SARS-CoV-2 ancestral strain-based vaccine with a 2-week apart. Booster doses were administered 4 months later with a 2-month interval (Fig. 1A). Neutralizing antibody titers against the ancestral strain and antigen-matched variants were evaluated 2 weeks after each booster dose, according to a pseudovirus (PsV) neutralizing assay.

Neutralizing activities against pre-Omicron and Omicron sublineages after booster immunization were significantly higher than those before booster shots (Fig. S1). For pre-Omicron variants (D614G and Beta PsVs), equivalent neutralizing titers were observed between one-dose and two-dose booster shots with each monovalent vaccine, with no substantive differences (Fig. 1B and C).

For Omicron sublineages, one- or two-dose pre-Omicron variant-based vaccines (Beta monovalent vaccine) elicited comparable neutralizing antibody titers against BA.5 (*P* = 0.2443), which were dramatically reduced compared with those against D614G and Beta (Fig. 1D), and had virtually no neutralizing capacity against BQ.1.1 or XBB.1 (Fig. 1E and F). By contrast, one booster dose of Omicron sublineage-based vaccines (BA.5, BQ.1.1 or XBB.1 monovalent vaccine) elicited more potent neutralizing responses against Omicron sublineage PsVs, with a further increase in neutralizing titers after the second booster dose (BA.5, 3.6 to 12.1-fold; BQ.1.1, 21.6 to 63-fold; and XBB.1, 11.2 to 141.5-fold) (Fig. 1D through F). Meanwhile, sera from mice vaccinated with BA.5 or XBB.1 exhibited a more pronounced neutralizing capacity against antigen-matched PsVs (Fig. 1D through F), while the BQ.1.1 monovalent vaccine evoked comparable immune responses against both BQ.1.1 and BA.5 (Fig. 1E).

In addition, one booster dose of each monovalent vaccine (Beta, BA.5, BQ.1.1, or XBB.1) elicited more pronounced immune responses against variants that emerged in the early stage of the pandemic than against variants that emerged subsequently. More potent neutralizing capacity against D614G PsV (NAT_50_: 23,896, 34,477, 15,904, 17,234, respectively) and Beta PsV (NAT_50_: 34,308, 42,316, 3,644, 8,807, respectively) was observed (Fig. 1B and C). There is a significant decline in neutralizing titers against variants antigenically distinct from ancestral strains, such as BQ.1.1 PsV (NAT50_50_: 60, 372, 494, 88, respectively) and XBB.1 PsV (NAT50_50_: 79, 109, 160, 190, respectively) (Fig. 1E and F). These findings indicated that immune responses following a single booster dose exhibited a strong bias toward earlier variants (D614G, Beta), with significantly reduced potency against antigenically distant Omicron sublineages (BQ.1.1, XBB.1). However, a two-dose booster regimen using Omicron (BA.5, BQ.1.1, or XBB.1) monovalent vaccines offered more potent cross-protective neutralizing capabilities against both ancestral and subsequent Omicron variants. This clearly suggested that a two-dose booster regimen with vaccines based on recently emerged variants effectively mitigated the prior immune bias and broadened the neutralizing antibody repertoire.

**Fig 2:**
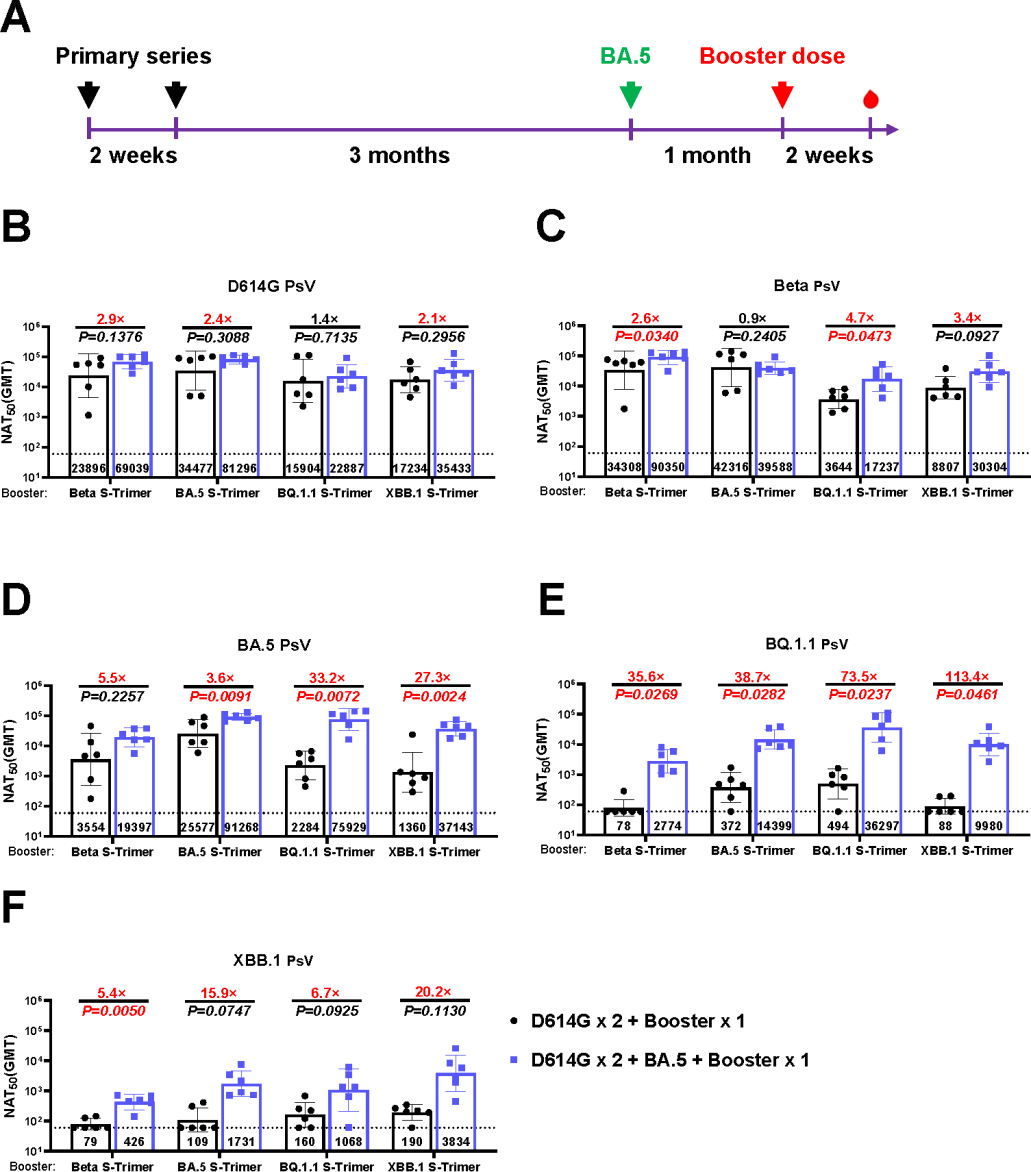
Humoral immune responses induced by boosting with a single-dose monovalent vaccine following priming series with or without BA.5 exposure in mice. (**A**) Schematic of the immunization design. Mice were intramuscularly administered two doses of D614G monovalent vaccine on Day 0 and Day 14. After 4 months, the monovalent vaccines (Beta, BA.5, BQ.1.1, or XBB.1) were injected into mice as a booster shot. Another group of priming mice was immunized with the BA.5-based vaccine, mimicking infection or vaccination with the BA.5 variant at 3 months and received a single booster shot with monovalent vaccines 1 month later. Serum neutralizing titers against PsV displaying D614G (**B**), Beta (**C**), BA.5 (**D**), BQ.1.1 (**E**), and XBB.1 (**F**). *P* values and fold change of neutralization were annotated above each bar, and geometric mean titers (GMTs) were labeled in the corresponding columns. Data were represented as mean ± SD, *n* = 6/group.

To investigate the influence of BA.5 breakthrough infection on immune responses evoked by variant-based booster doses, Balb/c mice were administered two doses of the D614G vaccine (2 weeks apart) to mimic the vaccination schedule of individuals who had previously received first-generation COVID-19 vaccines. Subsequently, a single dose of Beta, BA.5, BQ.1.1, or XBB.1 monovalent vaccine was administered as a booster after 4 months (D614G × 2 + Booster × 1). Before the administration of the booster dose, half of the animals were subjected to a one-dose BA.5 vaccine, mimicking infection or vaccination with the BA.5 variant (D614G × 2 + BA.5+ Booster × 1) (Fig. 2A).

After BA.5 exposure, an elevation of the neutralizing activity against various strains (D614G, Beta, BA.5, BQ.1.1, and XBB.1) was observed (Fig. S1 and S2). Compared with the D614G × 2 + Booster × 1 regimen, the D614G × 2 + BA.5 + Booster × 1 regimen induced slightly greater neutralizing responses against pre-Omicron variants (D614G and Beta) (Fig. 2B and C), and more potent neutralizing activities against BA.5 (3.6-fold to 33.2-fold), BQ.1.1 (35.6-fold to 113.4-fold), and XBB.1 (5.4-fold to 20.2-fold) (Fig. 2D through F).

Prior immunity bias was observed as implicated by limited neutralizing ability against the descendant Omicron subvariants (BA.5, BQ.1.1, XBB.1) in the D614G × 2 + Booster × 1 regimen. By contrast, as shown in Fig. 2D through F, an additional dose of BA.5-based vaccine (mimicking infection or vaccination with the BA.5 variant) before a booster dose mitigated immunological biases towards earlier variants, leading to enhanced neutralizing capacity against Omicron sublineages. Meanwhile, under the D614G × 2 + BA.5 + Booster × 1 regimen, neutralizing capacity against Omicron sublineages was comparable between boosters based on Omicron subvariants (BA.5, BQ.1.1, XBB.1) with a slightly higher neutralizing antibody titer against antigen-matched PsVs. Conversely, the booster dose based on pre-Omicron variants, such as the Beta monovalent vaccine, continued to demonstrate limited effectiveness against Omicron subvariants, reflecting the persistence of immunological biases, even after the additional dose of the BA.5 vaccine.

**Fig 3:**
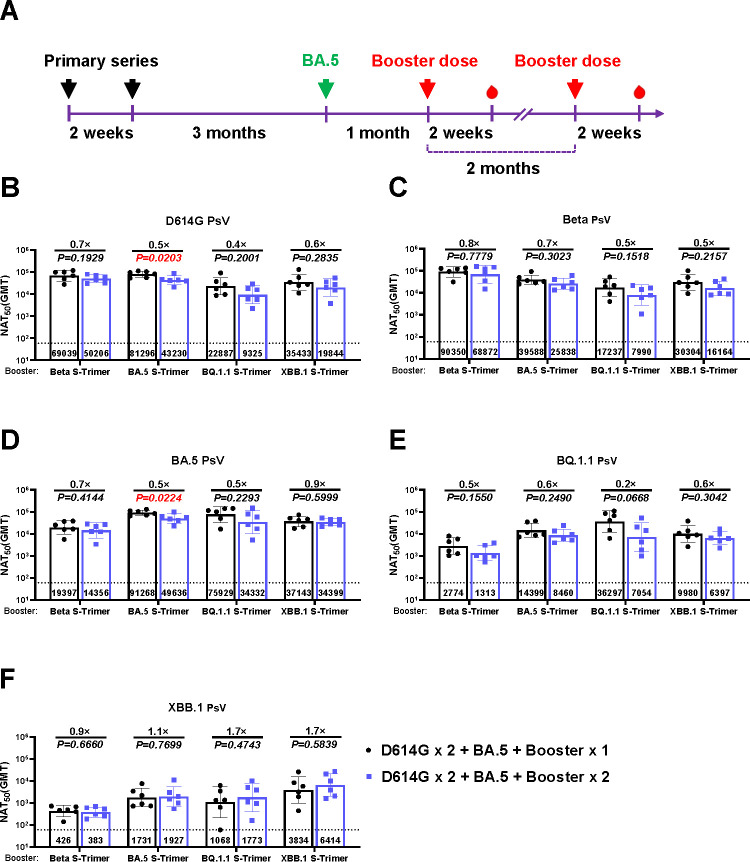
Neutralizing titers induced by boosting with one or two doses of monovalent vaccines following priming series and BA.5 exposure in BALB/c mice. (**A**) Schematic of the immunization design. Mice were intramuscularly administered two doses of the D614G monovalent vaccine as primary immunization over a 2-week interval. 3 months later, primed mice were immunized with BA.5-based vaccine, mimicking infection or vaccination with the BA.5 variant, and were boosted with one or two doses of monovalent vaccines at 1 and 2 months, respectively. Serum samples were collected at 2 weeks after the last vaccination for neutralization assays. Neutralizing activities against PsV displaying D614G (**B**), Beta (**C**), BA.5 (**D**), BQ.1.1 (**E**), and XBB.1 (**F**). *P* values and fold change of neutralization were annotated above each bar, and geometric mean titers (GMTs) were labeled in the corresponding columns. Data were represented as mean ± SD, *n* = 6/group.

We further explored the influence of an additional booster dose 1 month after the D614G × 2 + BA.5 + Booster × 1 regimen (Fig. 3A). Interestingly, in the context of BA.5 exposure, there was no notable disparity in the neutralizing capacity of either one or two booster doses against both the pre-Omicron variants (Fig. 3B and C) and descendant Omicron subvariants (Fig. 3D through F). These findings indicated that repeated booster immunization after BA.5 exposure did not further enhance immune responses, in sharp contrast with the D614G × 2 + Booster × 1 regimen (Fig. 1D through F).

### Neutralization of a single dose of XBB.1 monovalent vaccine and XBB.1-containing multivalent vaccine as a booster dose following ancestral strain-based primary series with subsequent BA.5 exposure

As of December 17, 2023, the primary series of COVID-19 vaccines has been administered to 67% of the global population, and over 90% of the total population has probably encountered infection by Omicron subvariants (25, 26). The selection of the antigen composition of COVID-19 vaccines as booster doses should take into account the potential for immune bias stemming from prior exposures.

**Fig 4:**
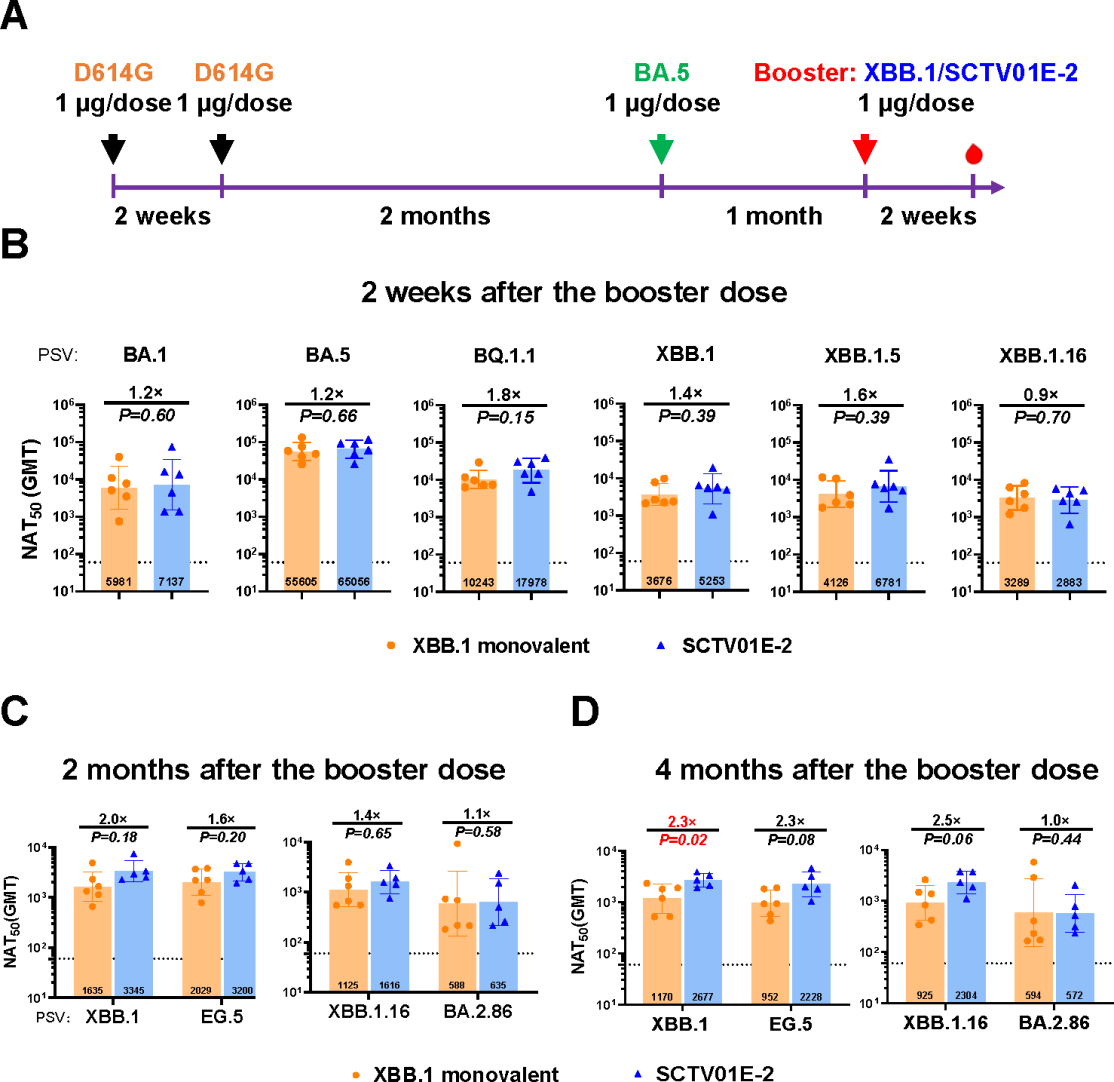
Neutralization capacities induced by a single booster dose of SCTV01E-2, or XBB.1 monovalent vaccines following priming series and BA.5 exposure in mice. (**A**) Schematic of the immunization design. Mice were intramuscularly administered two doses of the D614G monovalent vaccine as primary immunization over a 2-week interval. After 2 months, mice were immunized with the BA.5 vaccine (mimicking infection or vaccination with the BA.5 variant) and administered a single dose of XBB.1 monovalent vaccine or SCTV01E-2 1 month later. Serum samples were collected at 2 weeks, 2 months, or 4 months after the last vaccination for neutralization assays. (**B–D**) Neutralizing activity 2 weeks (**B**), 2 months (**C**), and 4 months (**D**) after the booster shot. *P* values and fold change of neutralization were annotated above each bar, and geometric mean titers (GMTs) were labeled in the corresponding columns. Data were represented as mean ± SD, *n* = 5-6/group.

As mentioned above, BA.5 exposure after the primary series attenuated the influence of immune bias (Fig. 2D through F). To clarify whether such kind of attenuation can be observed when multivalent vaccines, containing both earlier variants and XBB.1, are used as a booster dose, we compared the humoral immune responses evoked by an XBB.1 monovalent vaccine and a tetravalent vaccine based on trimeric spike protein of Beta, BA.1, BQ.1.1, and XBB.1 (named SCTV01E-2) in C57BL/6J mice. Animals were immunized with the BA.5 monovalent vaccine 3 months after 2-dose primary immunization. Subsequently, a booster dose of SCTV01E-2 or XBB.1 monovalent vaccine was administered 1 month later (Fig. 4A). Two weeks after the boosting immunization, sera were subjected to evaluation of neutralizing capacities against antigen-matched Omicron sublineages (BA.1, BQ.1.1, and XBB.1) and currently circulating Omicron sublineages (BA.5, XBB.1.5, and XBB.1.16). No discernible difference in neutralizing antibody titers was detected between SCTV01E-2 and XBB.1 monovalent vaccine groups against Omicron sublineages (Fig. 4B).

Furthermore, we evaluated the immune persistence of SCTV01E-2 and XBB.1 monovalent vaccine 2 months and 4 months after the booster dose against currently circulating variants XBB.1 and XBB descendant lineages (EG.5 and XBB.1.16), as well as BA.2.86, a variant under monitoring (VUM). 2 months after the booster dose, SCTV01E-2 and XBB.1 monovalent vaccine showed an inhibitory effect against XBB sublineages and BA.2.86 variants in contrast with the “adjuvant-only” group (Fig. S3). Compared to the XBB.1 monovalent vaccine, one booster dose of SCTV01E-2 induced comparable neutralizing antibody titers against XBB.1, EG.5, and XBB.1.16, with a slight increase by 2.0-fold, 1.6-fold, 1.4-fold, and 1.1-fold in GMT (Fig. 4C). These data indicated that SCTV01E-2 and XBB.1 monovalent vaccines exerted comparable and broad cross-protective potency against newly emerging Omicron strains. Neutralizing potency persisted for at least 4 months (Fig. 4D). Taken together, in the context of the primary vaccination series and BA.5 exposure, one booster shot of either SCTV01E-2 or XBB.1 monovalent vaccine showed significant cross-reactivity against newly emerging Omicron subvariants, alongside long-lasting immune responses. This implied that both the SCTV01E-2 tetravalent vaccine and the XBB.1 monovalent vaccine can effectively induce robust and broad immune responses against XBB or BA.2.86 descendant lineages in populations with prior immunity. Furthermore, it suggested that any potential immune bias resulting from prior exposure does not preclude the induction of effective immunity against these emerging variants by the booster vaccines.

### Neutralization of a single dose of XBB.1 monovalent vaccine and XBB.1-containing multivalent vaccine against the most recently emerged circulating variants in mice with prior exposure to previous dominant variants of SARS-CoV-2

**Fig 5:**
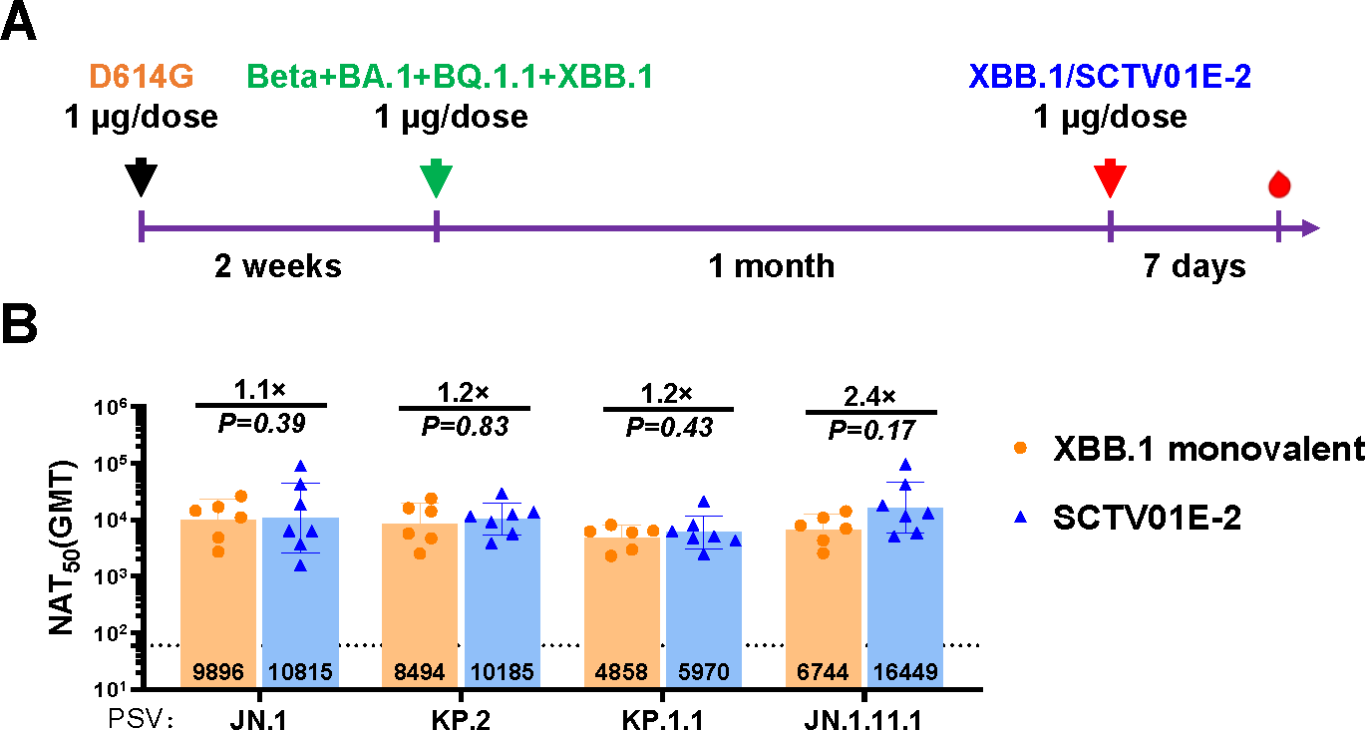
Neutralization capacities induced by a single dose of SCTV01E-2 or XBB.1 monovalent vaccine in mice with prior exposure to several previous dominant SARS-CoV-2 variants. (**A**) Schematic of the immunization design. Balb/c mice were intramuscularly administered one dose of the D614G vaccine (1 µg/dose). Two weeks later, a vaccine composed of S-Trimer of Beta, BA.1, BQ.1.1, XBB.1 (1 μg/dose) mimicking infection or vaccination with various dominant SARS-CoV-2 variants. Mice were subjected to a single dose of either XBB.1 monovalent vaccine or SCTV01E-2 one month later. Serum samples were collected 7 days later to measure the neutralizing titers. (**B**) Neutralizing activities against PsV displaying JN.1, KP.2, KP.1.1, and JN.1.11.1. *P* values and fold change of neutralization were annotated above each bar, and geometric mean titers (GMTs) were labeled in the corresponding columns. Data were represented as mean ± SD, *n* = 6-7/group.

JN.1 and its sublineages, KP.2, KP.1.1, and JN.1.11.1, which exhibit marked immune escape, have shown fluctuations in prevalence (27). To further investigate the effects of XBB.1 monovalent and multivalent vaccines on these newly prevalent strains, we conducted an analogous study to evaluate the neutralizing capacity against JN.1, KP.2, KP.1.1, and JN.1.11.1 of sera from mice with previous exposure to various dominant SARS-CoV-2 variants (Fig. 5A). Compared to the XBB.1 monovalent vaccine, a single dose of SCTV01E-2 induced comparable neutralizing antibody titers against JN.1, KP.2, KP.1.1, and JN.1.11.1, demonstrating slight increases of 1.1-fold, 1.2-fold, 1.2-fold, and 2.4-fold in GMT, respectively (Fig. 5B). These data indicated that SCTV01E-2 and XBB.1 monovalent vaccines exerted comparable and broad cross-protective potency against JN.1 and its sublineages, suggesting that both the SCTV01E-2 tetravalent vaccine and the XBB.1 monovalent vaccine could be effective in overcoming the influence of immune bias on vaccine efficacy against JN.1 and its sublineages in populations with previous exposure to various variants of SARS-CoV-2.

### Correlation analysis between pseudovirus and live virus neutralization activity of mouse sera

Due to the limitations of the Biosafety Level-3 Facilities and the restricted availability of serum, immune responses under each vaccination regimen were evaluated by pseudovirus neutralizing assay. Nevertheless, a large number of serological studies on SARS-CoV-2 have demonstrated a strong correlation between the results of SARS-CoV-2 pseudovirus neutralization activity and live virus microneutralization data, with a linear R2^2^ range of 0.385–0.993 (24, 28–30).

**Fig 6:**
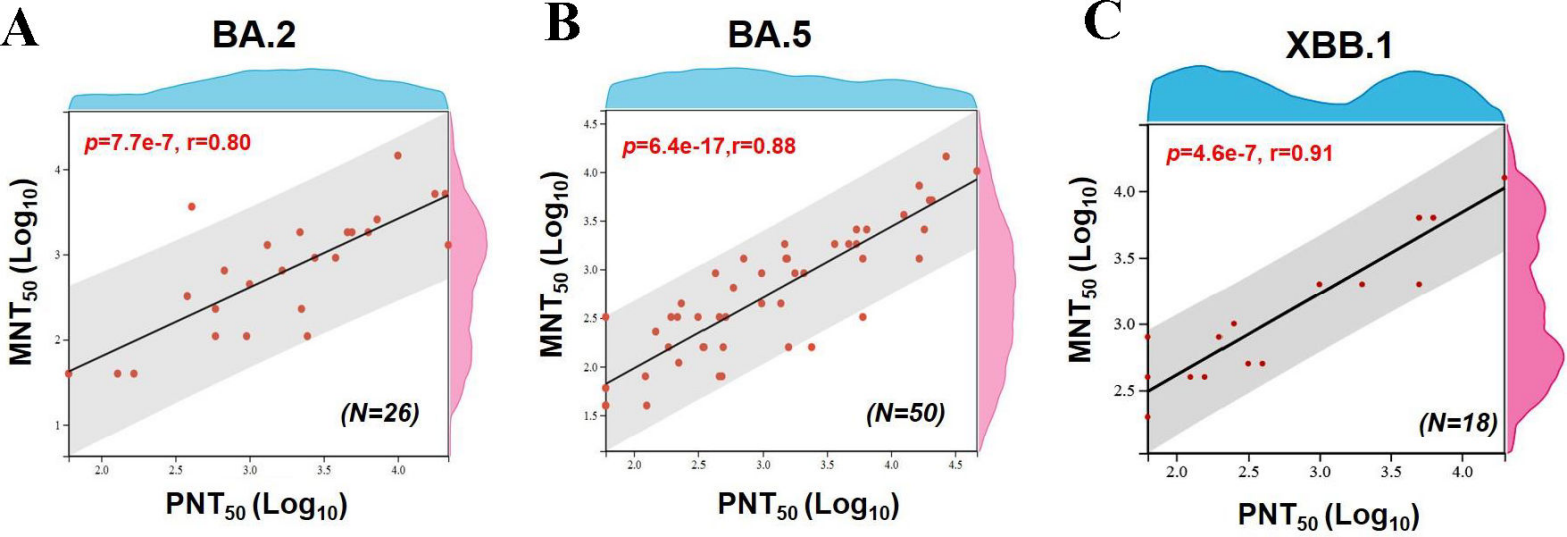
Correlation between pseudovirus and live virus neutralization activity of immune sera from C57BL/6 mice. Correlation analysis between serum neutralizing capability against live virus and pseudovirus of BA.2 (**A**), BA.5 (**B**), and XBB.1 (**C**) variants.

In addition, our team conducted a correlation analysis between live virus and pseudovirus neutralization activity of mouse sera against Omicron variants BA.2, BA.5, and XBB.1 live virus (micro neutralization test, MNT_50_) and pseudovirus (pseudovirus neutralization test, PNT_50_). MNT_50_ and PNT_50_ values for each variant were transformed to Log10. Person and Spearman correlation analyses were performed. The data indicated a significant positive correlation between PNT_50_ and MNT_50_ for the BA.2 (r = 0.80, *P* = 7.7e-7), BA.5 (r = 0.88, *P* = 6.4e-17), and XBB.1 (r = 0.91, *P* = 4.6e-7) variants (Fig. 6). These findings further substantiate the strong correlation between live virus and pseudovirus neutralization capacity.

## DISCUSSION

Vaccine failure refers to the reduction in vaccine efficacy against emerging variants due to repeated vaccination and exposure to evolving SARS-CoV-2 variants, which may pose a challenge to responding to the COVID-19 crisis (31–35). Therefore, it is necessary to clarify how variant strain-based SARS-CoV-2 vaccines affect the immune responses of individuals with divergent immune backgrounds to circulating variants, which may provide some guidance for the selection of antigen composition for updated COVID-19 vaccines against newly emerged circulating SARS-CoV-2 variants.

In May 2023, the Technical Advisory Group on COVID-19 Vaccine Composition (TAG-CO-VAC) recommended that antigens based on the Wuhan-Hu-1 strain should be excluded from the composition of future booster vaccines due to antigenic differences and immune bias from the original strain (2). In addition, immune bias could be modulated to some extent by the antigenic distance between the two strains (36, 37). The antigenic distance between SARS-CoV-2 variants and the ancestor D614G follows an ascending order of Beta, BA.5, BQ.1.1, and XBB.1 (6). Given that the antigenic distance of the original strain was closer to D614G and Beta, and farther from BQ.1.1 and XBB.1, a single booster dose of monovalent vaccines resulted in higher neutralizing titers against D614G and Beta, and lower titers against BQ.1.1 and XBB.1 (Fig. 1D through F), which is consistent with previous research results (38). Meanwhile, in the context of primary series, two doses of booster could not only significantly improve the neutralizing potency against antigen-matched emerging variants, but also enhance the immune responses against original strains, as demonstrated by both preclinical (8, 25, 39) and clinical (32, 40–44) studies. Consistent with previous studies, our data showed that two doses of Omicron boosters significantly potentiated neutralizing titers against BA.5, BQ.1.1, and XBB.1 strains while maintaining high neutralizing activity against the D614G variant (Fig. 1B through F).

In the context of vaccine-induced immunity, prior exposure to BA.5 attenuated the effect of immune bias on the neutralizing potency against Omicron subvariants (Fig. 1D through F), which is consistent with a previous study indicating that an additional dose of Omicron-based vaccines can potentiate the neutralizing capacity against Omicron subvariants in mice received a two-dose ancestral strain-based SARS-CoV-2 vaccine and one dose of Omicron-based vaccine (8). We explored whether a further booster dose could reinforce the immune response. As shown in Fig. 3, we did not observe a significant increase in neutralizing titers after the administration of another booster dose 2 months later, which implied that an immunologic plateau might have occurred, and the interval between each booster dose should be considered under vaccine-induced immunity.

This study compared the neutralizing potency between the XBB.1 monovalent vaccine and a tetravalent vaccine SCTV0E-2, in the context of multiple vaccination history (primary series and subsequent BA.5 exposure) against Omicron subvariants. Both SCTV01E-2 and XBB.1 monovalent vaccines were competent in inhibiting the original and newly circulating Omicron strains and attenuating the influence of immune bias (Fig. 4; Fig. S4). This phenomenon may be attributed to multiple factors, including the various vaccination histories, the time intervals between primary series and BA.5 exposure, as well as subsequent booster shots. In addition, the dosage of each vaccination and the types of vaccine administered each time can also modify the effect of immune bias (12, 14, 45–47). Meanwhile, clonal evolution of memory B cells (MBCs) contributes to the establishment of memory cells targeting a wider range of epitopes, which might dampen the probability of a robust response against the ancestral strains (36, 48). In addition, the FDA has made a recommendation of trivalent influenza vaccines in the 2024–2025 US influenza season (49). Compared with influenza viruses, a more rapid evolution rate has been observed in the spike protein of SARS-CoV-2 (50, 51). Uncertainty exists regarding the future dominant strain of SARS-CoV-2 and the selection of vaccine antigens (2, 38). Besides, the evolution of coronaviruses might lead to a weakening immunogenic response. Longitudinal analysis showed that the receptor-binding sites (RBS) of hCoV229E showed an overall attenuated antigenicity over time, while a significant reduction in Omicron RBS antigenicity and a decrease in neutralizing titers were also observed (52). Although our data did not reveal statistically significant differences in neutralizing antibody titers between the XBB.1 monovalent vaccine and the multivalent SCTV01E-2 vaccine (Fig. S4), the broader epitope coverage of multivalent vaccines may theoretically enhance the magnitude and breadth of cross-neutralization responses, thereby providing a buffer for unpredictable future variants. However, this hypothesis necessitates rigorous validation through larger clinical trials and real-world effectiveness studies, particularly given the rapid evolution of SARS-CoV-2.

In summary, our data elucidated the characteristics of humoral immune responses of SARS-CoV-2 variant-based vaccines as booster doses in the context of various immunity history in mice. It is crucial to determine the optimal immunization regimens and update the vaccine components to address the risk posed by the continuously evolving SARS-CoV-2 variants. Our findings confirmed the existence of immunological biases toward earlier variants, the influence of which might be attenuated by exposure to Omicron before a booster dose. Furthermore, different vaccination schedules can exhibit distinct neutralizing capacities, which should be taken into account when developing novel vaccines and immunization regimens.

## SUPPLEMENTAL MATERIAL

Data Set S1 spectrum.02907-24-s0001.xlsx Raw data for NAT_50_.

Supplemental figures spectrum.02907-24-s0002.docx Figures S1 to S4.

ASM does not own the copyrights to Supplemental Material that may be linked to, or accessed through, an article. The authors have granted ASM a non-exclusive, world-wide license to publish the Supplemental Material files. Please contact the corresponding author directly for reuse.
