# Supplementary material for: Immunogenicity of monovalent and multivalent subunit vaccines against SARS-CoV-2 variants in mice with divergent vaccination history
Source: Microbiol Spectr. 2025 Jul 17;13(8):e02907-24. doi: 10.1128/spectrum.02907-24 (PMC12323355; doi:10.1128/spectrum.02907-24)
Supplement: Supplemental figures — Figures S1 to S4. [file spectrum.02907-24-s0002.docx]

**Supplementary Data**


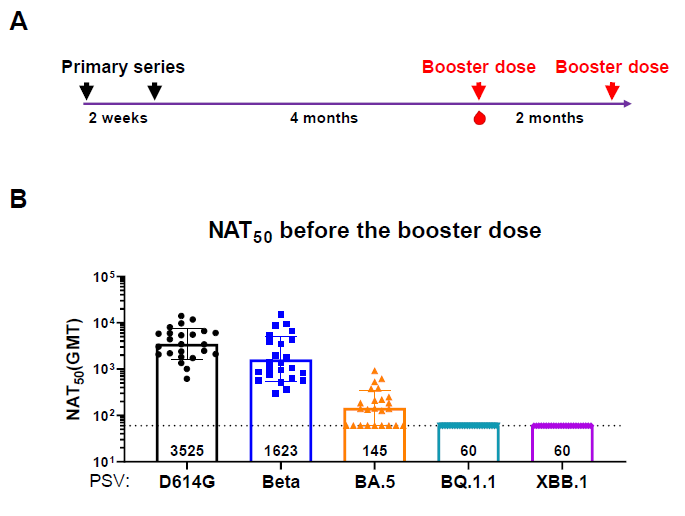


**Figure S1. Neutralizing antibody responses before booster shots in mice received prior 2-dose primary series.** (**A**) Schematic of the immunization design. Mice were intramuscularly administrated with two doses of D614G monovalent vaccine on Day 0 and Day 14. After 4 months, serum samples were collected to assess neutralization activity prior to the administration of booster shots, which were conducted on the same day as serum collection. (**B**) Serum neutralizing titers before booster shots against PsV displaying D614G, Beta, BA.5, BQ.1.1, and XBB.1. Data were represented as mean ± SD, n = 24/group.


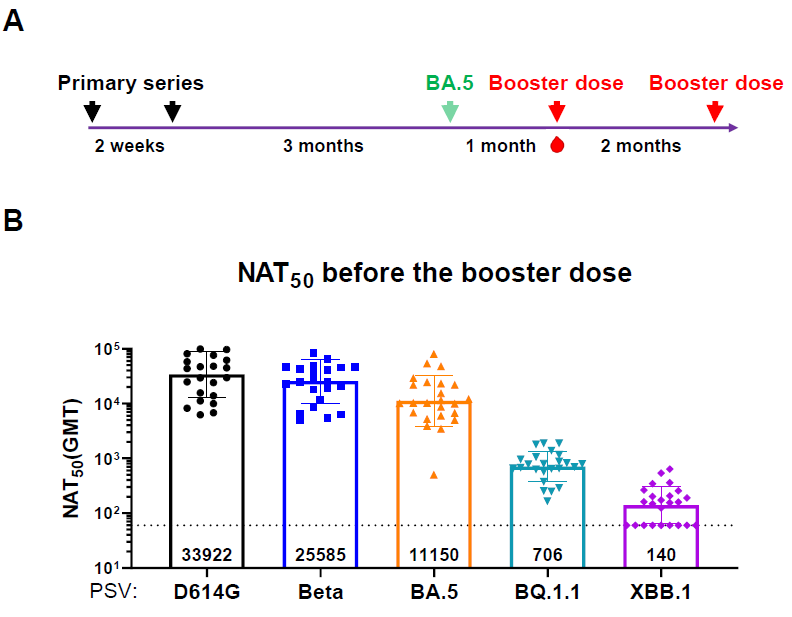


**Figure S2. Neutralizing antibody titers before booster shots in mice received prior 2-dose primary series and 1-dose BA.5 exposure.** (**A**) Schematic of the immunization design. Mice were intramuscularly administrated with D614G monovalent vaccine as primary immunization over a 2-week interval. 3 months later, priming mice were infected with the BA.5 variant. After 1 month, serum samples were collected to assess neutralization activity prior to the administration of booster shots, which were conducted on the same day as serum collection. (**B**) Serum neutralizing titers before booster shots against PsV displaying D614G, Beta, BA.5, BQ.1.1, and XBB.1. Data were represented as mean ± SD, n = 24/group.

**
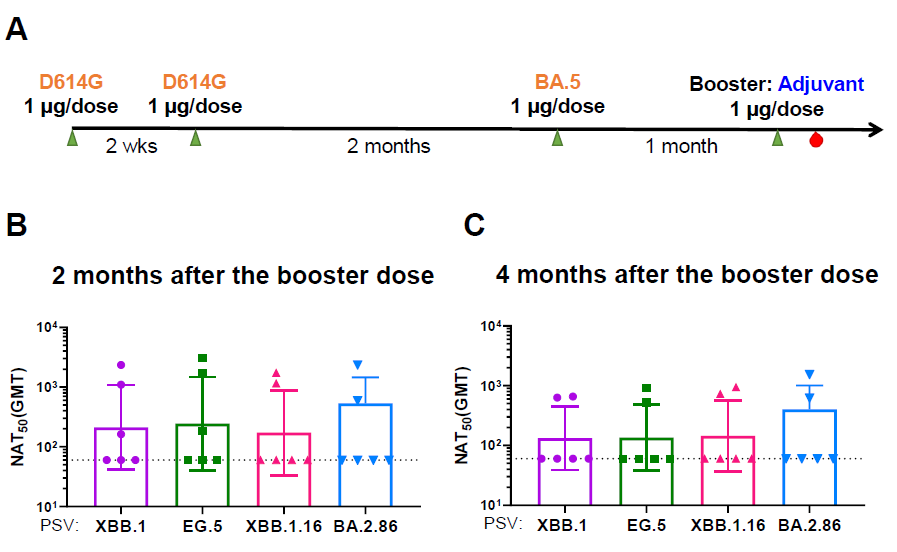
**

**Figure S3. Neutralization capacities of sera from mice boosted with adjuvant only following 2-dose primary series and 1-dose BA.5 exposure.** (**A**) Schematic of the immunization design. Mice were intramuscularly administrated with two doses of D614G monovalent vaccine as primary immunization over a 2-week interval. After 2 months, mice were infected with the BA.5 strain and boosted with a single dose of adjuvant 1 month later. Serum samples were collected at 2 months or 4 months after the last vaccination for neutralization assays. (**B**) Neutralizing activity of booster shots after 2 months. (C) Neutralizing activity of booster shots after 4 months. Data were represented as mean ± SD, n = 6/group.


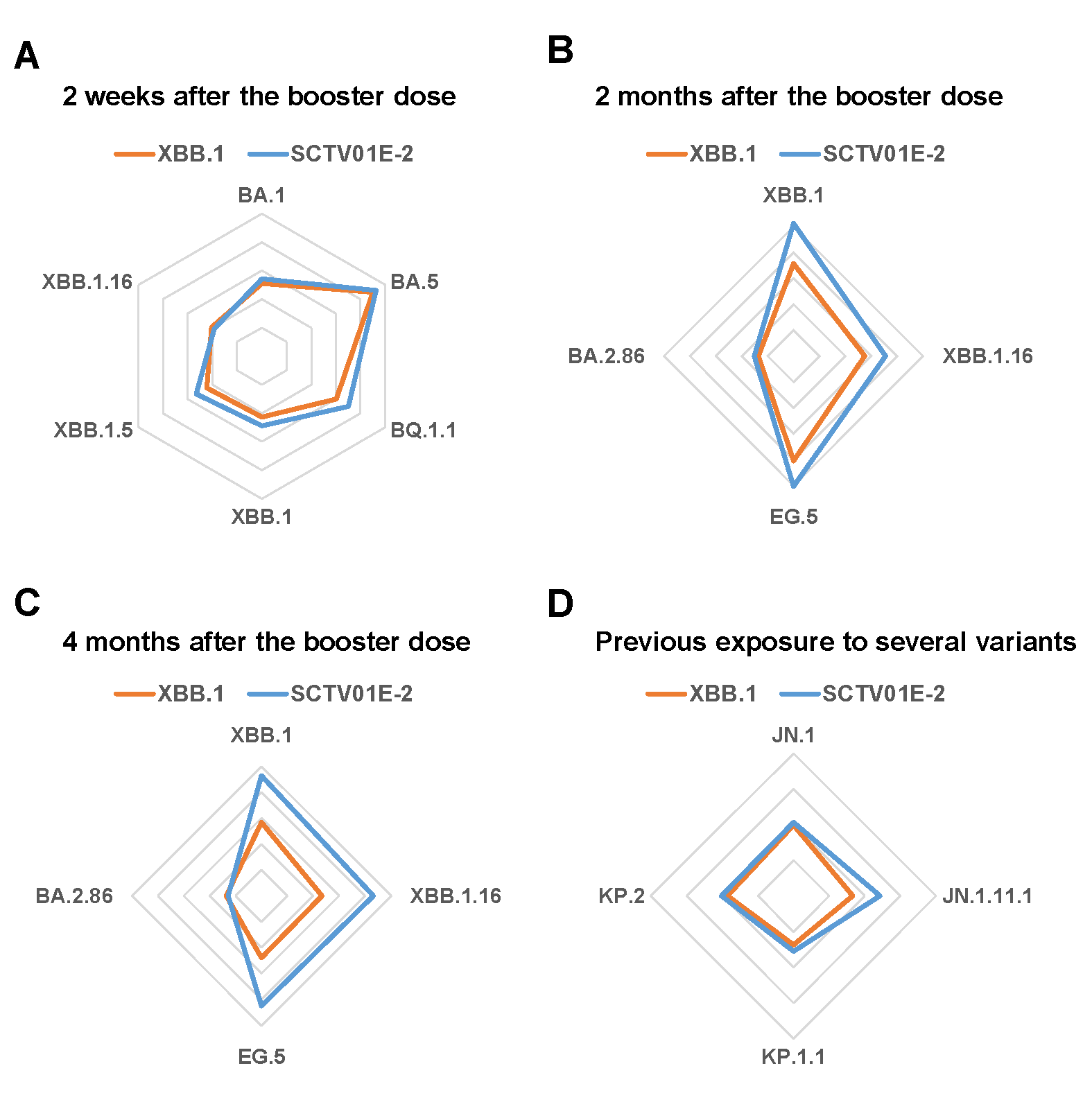


**Figure S4. The radar charts of neutralizing response tropism induced by XBB.1 monovalent vaccine and SCTV01E-2 in mice.** Data were converted from GMTs values to Log10 values for further analysis. (**A-C**) Neutralizing activity 2 weeks, 2 months, and 4 months after the booster shot in mice with 2-dose priming series and 1-dose BA.5 exposure (Data originated from Figure 4B-D). (**D**) Neutralizing activity in mice with prior exposure to several previous dominant SARS-CoV-2 variants (Data originated from Figure 5B). Data were represented as mean ± SD.
